# Impact of long COVID on the heart rate variability at rest and during deep breathing maneuver

**DOI:** 10.1038/s41598-023-50276-0

**Published:** 2023-12-20

**Authors:** Andréa Lúcia Gonçalves da Silva, Luana dos Passos Vieira, Luiza Scheffer Dias, Cecília Vieira Prestes, Guilherme Dionir Back, Cassia da Luz Goulart, Ross Arena, Audrey Borghi-Silva, Renata Trimer

**Affiliations:** 1 Department of Health Sciences, University of Santa Cruz do Sul, Rio Grande do Sul, Brazil; 2 Nucleus of Research in Surveillance, Prevention and Rehabilitation in Cardiorespiratory Diseases, Rio Grande do Sul, Brazil; 3 Scientific Initiation Scholarship at Laboratory of Cardiorespiratory Rehabilitation, University of Santa Cruz do Sul, Rio Grande do Sul, Brazil; 4 Cardiopulmonary Physiotherapy Laboratory, Physiotherapy Department, Federal University of São Carlos (UFSCar), São Carlos, São Paulo, Brazil; 5 Department of Physical Therapy, College of Applied Health Sciences, University of Illinois Chicago, Chicago, IL USA

**Keywords:** Cardiology, Diseases

## Abstract

While the majority of individuals with coronavirus disease 2019 (COVID-19) recover completely, a significant percentage experience persistent symptom, which has been characterized as Long COVID and may be associated with cardiac and autonomic dysfunction. We evaluated heart rate variability (HRV) at rest and during deep-breathing (M-RSA) in patients with Long COVID. Case–control design involved 21 patients with Long COVID and 20 controls; the HRV was evaluated (POLAR system) at rest in the supine position and during M-RSA and expressed in time domain and non-linear analysis. In the supine position we found a reduction HRV measures in Long COVID’ patients compared to controls for: Mean_iRR (*p* < 0.001), STD_iRR (*p* < 0.001); STD_HR (*p* < 0.001); SD1 (*p* < 0.001); SD2 (*p* < 0.001); alpha2 (*p* < 0.001). In the M-RSA we found reduction Mean_iRR (*p* < 0.001), STD_iRR (*p* < 0.001), STD_HR (*p* < 0.001), rMSSD (*p* < 0.001), RR_tri-index (*p* < 0.001) in Long COVID’ patients except for highest Mean_HR *p* < 0.001. In conclusion, Long COVID reduced HRV at rest and during deep breathing. These findings may imply impairment of cardiac autonomic control when symptoms of COVID-19 persist following initial recovery.

## Introduction

The coronavirus disease 2019 (COVID-19) has had a massive effect on the world’s health, societal function, and economy. The COVID-19 pandemic caused high morbidity and mortality worldwide, currently resulting in > 500 million people infected and > 6 million deaths; in Brazil alone > 30 million people have been infected and > 600,000 have died^1^. While most people with COVID-19 recover completely, others continue to experience chronic and diverse symptoms that have been characterized as Long COVID^2^. The term “Long COVID” describes the long-lasting effects of COVID-19 which was categorized into two stages depending on the duration of prolonged symptoms: (1) ‘post-acute COVID’ which includes those cases in which symptoms persist > 3 weeks but are < 12 weeks; and (2) ‘chronic COVID’ in which symptoms persist > 12 weeks^3^. After the acute impacts of COVID-19 were the initial focus of concern and were widely researched and published, it is increasingly clear that many patients continue to experience a myriad of symptoms consistent with acute phase infection and/or some new chronic symptoms^4^, such as heart palpitations, fatigue, orthostatic intolerance, dizziness, brain fog, nausea, anxiety, hyperhidrosis and syncope^5^. COVID-19 is also associated with myocardial injury and subsequent cardiac dysfunction can cause cardiac arrhythmias^6^. Among patients hospitalized with acute COVID-19 infection, palpitations remain a common symptom among the much larger outpatient population of COVID-19 survivors in the convalescent stage of the disease^7^.

Studies have also revealed that many patients developed postural orthostatic tachycardia syndrome (POTS) following COVID-19 infection^2,8,9^ and in hospitalized COVID-19 patients with mild to moderate symptoms, parasympathetic dominance has been demonstrated compared to healthy controls^10^. In another study analyzing heart rate variability (HRV) in critically ill COVID-19 patients, the presence of autonomic imbalance with predominance of the parasympathetic system due to sympathetic tone depletion was demonstrated^11^. In this context, an autonomic imbalance precipitated by COVID-19 infection increases sympathetic tone acutely and may prevent parasympathetic overshooting^12^. Dysautonomia, characterized by dysregulation of HRV, may explain the persistent symptoms observed in Long COVID patients^13^; there is currently a lack of evidence demonstrating how long these autonomic symptoms persist post infection^9^. Moreover, there are currently no studies evaluating the respiratory sinus arrhythmia maneuver (M-RSA) in patients with Long COVID. During M-RSA, patients are induced with parasympathetic stimulus and this response may be associated with changes in both lung compliance and lung stretch reflex responses in patients with cardiopulmonary disease and in healthy humans. Respiratory sinus arrhythmia is very important to assess the impaired cardiac autonomic control^14^.

In this respect, to analyze the status of the autonomic nervous system (ANS) by measuring HRV in Long COVID patients referred for rehabilitation programs is needed^15^. Therefore, the main objective of the current study was to evaluate HRV in supine position and during the M-RSA in patients with Long COVID. We hypothesize that patients with Long COVID present altered HRV responses at rest and during M-RSA.

## Results

Baseline patient characteristics are shown in Table 1. Long COVID patients were older and had a higher body mass index (BMI) compared to the control group. However, both groups were similar regarding Caucasian ethnicity.

**Table 1:** Clinical characteristics of the patients when they joined LARECARE.

Variables	Long COVID (n = 21)	Control group (n = 20)	*p* value
Age, yr	53.9 ± 11.1	40.0 ± 12.6	0.001
Caucasian ethnicity, n (%)	21 (100)	16 (80)	0.620
Male, n (%)	13 (61.9)	11 (55)	0.653
BMI (kg/m^2^)	28.6 ± 5.2	24.8 ± 3.2	0.009
Smoking, n (%)			
Never smoked	14 (66.7)	19 (95)	
Ex smoked	6 (28.6)	1 (5)	
Smoker	1 (4.8)		
Hospitalized, (n = 17)		NA	
Length of stay (days)	9.5 (5–41)	NA	
Weight loss (kg)	7.4 ± 3.7	NA	
% Pulmonary involvement by CT		NA	
≤ 50%	9 (42.9)	NA	
≥ 50%	8 (38.1)	NA	
Persistent symptoms after COVID-19 infection, n (%)			
Fatigue	15 (71.4%)	NA	
Dyspnea	5 (23.8%)	NA	
Cough	4 (19%)	NA	
Medications, n (%)			
Oral bronchodilator	2 (9.5)	NA	
Inhaled bronchodilator	3 (14.3)	NA	
Corticosteroid	4 (19.0)	NA	
Antidepressant	4 (19.0)	NA	
Antihypertensive	6 (28.6)	NA	
Diuretic	3 (14.3)	NA	
Anticoagulant	6 (28.6)	NA	
Statin	4 (19.0)	NA	
Anti-inflammatory	2 (9.5)	NA	

Data expressed on average ± standard deviation; n: sample number; BMI: Body mass index; kg/m^2^: kilogram per square meter; NA: not applicable; CT: computed tomography.

In the time domain index of HRV analysis, Mean iRR, STD iRR and STD HR were significantly lower in the Long COVID group. In contrast, mean HR was significantly higher in the Long COVID group compared to controls (Table 2). Non-linear HRV analysis by SD1, SD2 and alpha2 were significantly lower in the Long COVID, except alpha1 was significantly higher in the Long COVID group.

**Table 2:** Heart Rate Variability during the at rest-supine position in the Long COVID and Control groups.

Variable	Long COVID (n = 21)	Control group (n = 20)	*p* value
	Median (Q1–Q3)	Median (Q1–Q3)	
HRV, time domain			
Mean iRR (ms)	722.8 (663.6–789.5)^#^	964.6 (908.3–1014.5)	< 0.001
STD iRR (ms)	14.7 (5.4–17.6)	42.1 (29.5–51.5)	< 0.001
Mean HR (bpm)	83.0 (76.1–90.5)	62.6 (59.2–66.1)	< 0.001
STD HR (bpm)	1.7 (1.0–2.6)	3.1 (2.1–3.8)	< 0.001
rMSSD (ms)	10.2 (3.4–13.3)	38.1 (27.4–49.9)	< 0.001
RR tri index	3.3 (1.8–4.9)	11.1 (7.9–13.1)	< 0.001
HRV, non-linear			
SD1 (ms)	7.2 (2.4–9.4)	27.0 (19.4–36.9)	< 0.001
SD2 (ms)	18.9 (7.0–22.8)	54.7 (35.7–65.5)	< 0.001
*ApEn*	1.0 (0.9–1.0)	0.9 (0.8–1.0)	0.340
SampEn	1.5 (1.4–1.8)	1.6 (1.3–1.7)	0.938
Alpha 1	1.2 (1.0–1.3)	0.9 (0.8–1.1)	0.003
Alpha 2	0.5 (0.4–0.6)	0.7 (0.5–0.8)	0.006

HRV, Heart Rate Variability; iRR, RR interval time series in milliseconds (ms); Mean iRR, mean RR interval of the series; STD iRR, standard deviation RR interval; Mean HR, mean heart rate in beats per minute (bpm); STD HR, standard deviation of heart rate mean; rMSSD, root mean square sum of squares of the differences between the RR intervals; RR tri index, triangular index; SD1,standard deviation measuring the dispersion of points in the plot perpendicular to the line of identity; SD2, standard deviation measuring the dispersion of points along the line of identity; ApEn, Approximate Entropy; SampEn, Sample Entropy; Q1, 25 percentile–Q3, 75 percentile.

In relation to M-RSA responses, the Long COVID demonstrated significantly lower values for all time indexes, with the exception of mean HR, when compared to controls (Table 3).

**Table 3:** Behavior of the Heart Rate Variability of the evaluated patients during M-RSA.

Variable	Long COVID (n = 21)	Control group (n = 20)	*p* value
	Median (Q1–Q3)	Median (Q1–Q3)	
HRV, time domain			
Mean iRR (ms)	675.7 (624.7–778.8)	866.3 (758.2–927.0)	< 0.001
STD iRR (ms)	20.4 (14.9–31.9)	63.0 (53.6–101.1)	0.006
Mean HR (bpm)	88.9 (78.2–96.2)	69.7 (65.0–80.2)	< 0.001
STD HR (bpm)	2.9 (2.3–4.3)	6.0 (4.4–6.8)	< 0.001
rMSSD (ms)	14.3 (7.8–19.4)	42.6 (26.5–55.4)	< 0.001
RR tri index	5.4 (3.7–8.1)	14.6 (12.2–17.6)	< 0.001

HRV, Heart Rate Variability; iRR, RR interval time series in milliseconds (ms); Mean iRR, mean RR interval of the series; STD iRR, standard deviation of RR interval; Mean HR, mean heart rate in beats per minute (bpm); STD HR, standard deviation of heart rate means; rMSSD, root mean square sum of squares of the differences between the RR intervals; RR tri-index, triangular index; Q1, 25 percentile–Q3, 75 percentile.

## Discussion

The main findings of the current study were as follows: (1) at rest, patients with Long COVID presented altered time and non-linear index of HRV suggesting high HR, lower parasympathetic tone (rMSSD; SD1), lower variability of RR intervals (RR tri index) and total variability (SD2) as well as impaired fluctuation analysis with increased risk of sudden death (alpha 1) compared to controls; (2) patients with Long COVID responded to the M-RSA with an increase in time index of HRV, however this response were significantly lower compared to controls, reinforcing lower parasympathetic tone (rMSSD), lower variability of RR intervals (RR tri index) and lower HR dynamics.

Different methods of autonomic assessment have been used in contemporary literature (pupillometry, microneurography, negative lower body pressure, cold pressor test, Ewing protocol, Holter, etc.) however, their use in clinical settings is limited^16^. To our knowledge, no studies with Long COVID patients have investigated heart rate variability in different postures and M-RSA with the device Pro-Trainer 5 with POLAR software, an accessible tool both in terms of cost and practicality, in addition to presenting good accuracy both at rest and during exercise^17^. Our study is the first to use the Pro-Trainer and this is of great clinical relevance in the context of diagnosing autonomic dysfunction and cardiovascular risk.

Research has demonstrated COVID-19 not only affects the cardiovascular system in its acute phase but can have prolonged negative effects^4^. Most patients with COVID-19 recover completely without sequela, while some patients continue to have diverse symptoms, including autonomic dysfunction, for longer than 12 weeks without an alternative diagnosis^12,18^. Long COVID symptoms of inappropriate palpitations, fatigue, orthostatic intolerance, dizziness, brain fog, nausea, anxiety, hyperhidrosis and syncope have been reported; there is currently a lack of evidence to indicate how long these autonomic symptoms will last^9^.

The mechanism of dissemination and development of COVID-19 in the human body, the immune system response, and the action of the virus on the Autonomic Nervous System (ANS), are obscure topics. Infection by the COVID-19 virus can generate inflammatory responses, once the immune system is active, which can lead to systemic damage^19^ including autonomic dysfunction mediated by the virus. Some studies have reported the association between autonomic dysfunction, neurotropism, hematogenous route or neuronal dissemination^20,21^. In this sense, the conflicting results in the current scenario refers to the increase in sympathetic activity at rest, which can lead to premature death^22^; contrasting with in young males and adult patients after COVID-19 showing increase of the parasympathetic nervous system activity demonstrating increased HRV indices than controls^18^. The findings of the current study differ from this concern [i.e., high HR, lower parasympathetic tone (rMSSD; SD1) and lower RR tri index and total variability (SD2) and increased risk of sudden death (alpha 1)]. We hypothesize that the differences found in our study are related to the clinical characteristics of the sample in relation to the literature [i.e., restricted to young men and/or young adults with a predominance of females, non-hospitalized and/or non-symptomatic patients^12,18^. These alterations in HR modulation trigger the emergence of cardiovascular diseases and inadequate adaptations of the ANS^23^, altering the homeostatic state of the body.

In this sense, patients with Long COVID may present with dysautonomia characterized by an imbalance of HRV, which is reflected in the band potencies of 0.15–0.4 Hz^13^, and highlights that this dysautonomia could explain the persistent symptoms observed in patients with Long COVID^13^. In our study, we observed similar results with a lower complexity of HRV in patients with Long COVID [lower variability of RR intervals (RR tri index) and total variability (SD2) and impaired fluctuation analysis with increased risk of sudden death (alpha 1)] compared to controls.

In a Mayo Clinic study including patients with symptoms related to postinfectious autonomic dysfunction after COVID-19, 63% were found to have abnormal findings on standardized tests of autonomic function, such as cardiovagal function analyzing heart rate responses to deep breathing and the Valsalva maneuver^12^. The most common post-COVID-19 autonomic manifestation was orthostatic intolerance, and the remaining changes ranged from symptomatic postural orthostatic tachycardia to severe autonomic dysfunction^24^. Our observation and clinical data suggest that Long COVID patients have the highest Mean HR in the first few days and weeks of the convalescent phase. Regarding M-RSA, our findings suggest that patients after hospitalization had a worse cardiac autonomic modulation, with lower parasympathetic tone (rMSSD), lower variability of RR intervals (RR tri index) and lower HR dynamics. Autonomous innervation is the primary extrinsic control mechanism that regulates HRV and cardiac performance. Thus, this autonomic dysregulation likely represents the cause and effect of the different stages of COVID-19, the severe inflammatory system response syndrome (SIRS)^11^ and its compensatory anti-inflammatory response until Long COVID which can be influenced by frequency and depth of breathing. Many patients have restrictive pulmonary conditions after infection, but only 15% have restrictive and obstructive patterns^25^, and these changes can directly influence the ability to perform M-RSA. We highlight that M-RSA was performed in a controlled environment with a metronome, where there is control of the RR and the stimulus for deep breathing, providing good capacity to evaluate the parasympathetic modulation response.

We found that HRV is markedly impaired in the presence of Long COVID. Therefore, strategies aimed at improving autonomic control index can improve the cardiovascular risk in these patients, as well as symptomatological and pulmonary function changes, since the cardiorespiratory interaction is closely associated with peripheral oxygen supply and symptoms of persistent fatigue. These results, therefore, emphasize the multifactorial nature of the cardiopulmonary impairment present in these patients, associated with the symptomatological manifestations (i.e., dyspnea and feeling of fatigue) present in these patients. Our findings provide a rationale for improving fatigability with interventions aimed at improving cardiac and respiratory system autonomic control, through pharmacological and non-pharmacological measures aimed at reducing the cardiovascular risk in these patients. For example, cardiorespiratory rehabilitation can reduce muscle fatigue and improve cardiac autonomic function in other chronic conditions and has been the focus of numerous studies in patients with Long COVID. Future clinical trials on the impact of these interventions on cardiocirculatory and autonomic responses should look more carefully at the relevance of peripheral and respiratory muscle performance enhancing cardiac autonomic control in this patient population.

This was a single-center study with a small sample size. Asymptomatic patients with COVID-19 were not included. Patients' breathing characteristics and drug treatment were not measured and standardized in this study and the patients were referred to LARECARE in different times after COVID-19 infection. The differences in age and BMI between the Long COVID and control group can be considered a confounding factor, so we performed a linear regression analysis to verify the influence of both variables on the HRV indexes and no significant difference was found, demonstrating that age did not influence the main outcome of the study. Moreover, there was no follow-up of the patients over time to verify whether or not HRV dysfunction persists.

## Conclusion

In this study, patients with Long COVID presented with abnormal autonomic tone, represented by depressed HRV by time domain (lower parasympathetic tone; lower variability of RR intervals) as well as reduced complexity. These findings may represent a potential risk of sudden death. Finally, poor HRV during a deep-breathing maneuver was observed in these patients. Strategies focused on improving these responses through pharmacological and non-pharmacological interventions in patients who have Long COVID symptoms deserve further investigation.

## Methods

### Study design and participants

The present study prospectively selected 71 volunteer participants, from December 2020 to January 2022: 51 patients with a COVID-19 diagnosis and 20 control participants without a COVID-19 diagnosis. This study was designed according to the recommendations of the STROBE statement, and it was conducted at the Cardiorespiratory Rehabilitation Laboratory (LARECARE) at Santa Cruz Hospital in Rio Grande do Sul, Brazil. The study protocol was registered at the University of Santa Cruz do Sul (UNISC) and complies with the Declaration of Helsinki for research involving human subjects, as approved by the Research Ethics Committee of the University of Santa Cruz do Sul under protocol number 5,194,614, and written informed consent was obtained from all participants.

**Figure 1:**
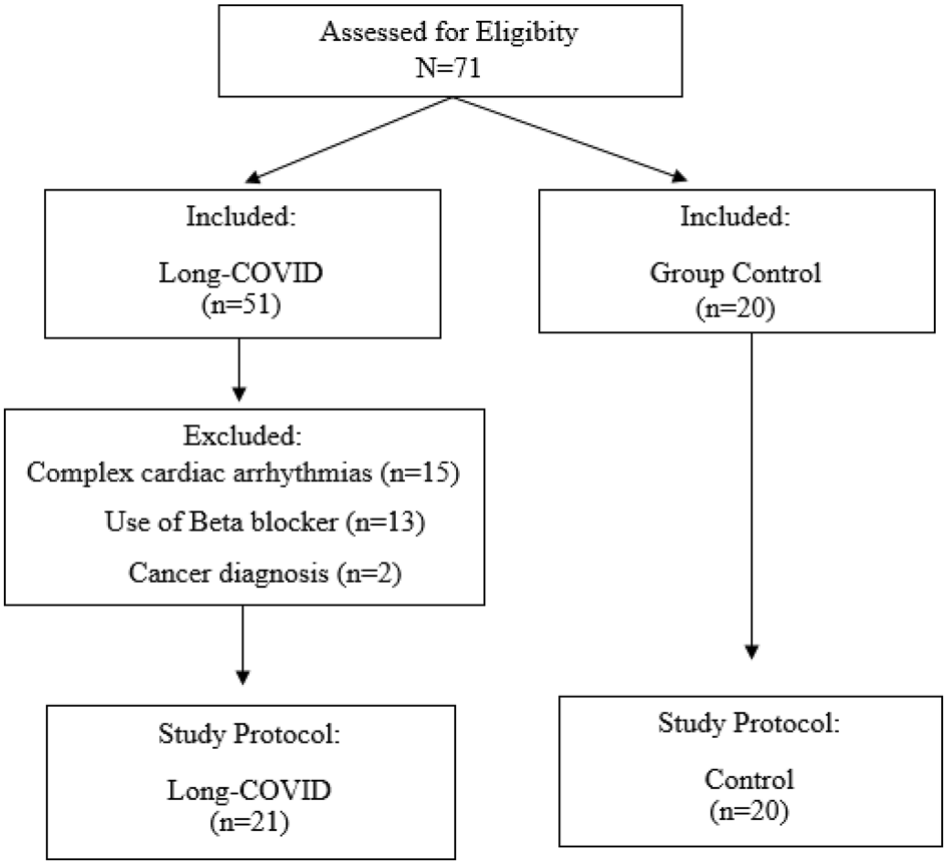
Flow chart of study population selection. Seventy-one patients with Long COVID from LARECARE were recruited. Thirty patients did not fulfill the inclusion criteria: complex cardiac arrhythmias (n = 15); use of Beta blocker (n = 13); and Cancer diagnosis (n = 2). Included in the study 21 patients with Long COVID and 20 controls subjects.

Convenience sampling consisted of patients referred to LARECARE by a physician due to prolonged respiratory and physical symptoms 4–16 weeks post COVID-19 infection, which was confirmed by positive polymerase chain reaction test, serologic test or a computed tomography (CT) scan. We included males and females undergoing treatment at LARECARE, with preserved cognitive ability, who agreed to participate in this research and signed an informed consent. Exclusion criteria were complex cardiac arrhythmias (e.g. atrial fibrillation and left bundle branch block by ECG WINCARDIO USB—Version 11.1.0.0), unstable angina, use of beta blockers and a cancer diagnosis (Fig. 1). The control group (CG) consisted of healthy subjects who sought the aforementioned health services for suspected disease and tested negative for COVID-19, according to WHO guidelines. After a negative diagnosis, volunteers without comorbidities, who did not use controlled medications, non-smokers, and matched by sex were selected for the CG.

### Data collection

Every patient completed a comprehensive evaluation process over three days: (1) clinical evaluation by a physical therapist; (2) resting electrocardiogram to rule out the presence of cardiac arrhythmias; (3) intervals R-R (iRR) and HR assessment in the supine position and M-RSA.

### Measurements

#### Heart rate variability

All subjects were evaluated during the same period of the day (in order to avoid differences in response due to circadian rhythm) at an experimental room temperature (22–24° C) and relative air humidity (50–60%) controlled. Subjects were instructed to abstain from caffeinated and alcoholic beverages and not to perform exercise on the day before data collection. The individuals were instructed not to speak during the collection, as well as not to sleep. Initially, the individuals were positioned supine and after a rest period of 15 min, data collection was started. Heart Rate (HR) recording was performed using a heart rate monitor (POLAR system S810i, Kempele, Finland), where an elastic strap (POLAR T31 transmitter) was positioned around the trunk of each subject at the level of the lower third of the sternum. The signals were continuously transmitted to the receiving unit through an electromagnetic field (watch placed on the right wrist), and all data were later transferred to a computer using the Pro-Trainer 5 POLAR software. The RR interval (RRi) was registered using the POLAR system at rest in the supine (10 min) position, as well as during M-RSA (4 min, in the seated position). In this maneuver, the researchers instructed the subjects to perform a sequence of deep, slow inhalations and exhalations. Each respiratory cycle lasted 10 s (5 s of inspiration, 5 s of expiration). The subjects followed the researchers' verbal commands to maintain a respiratory rate of six breaths per minute, which is expected to induce maximum respiratory sinus arrhythmia. The iRR of the respiratory sinus arrhythmia (RSA) test was analyzed using commercial software (MATLAB R2019a, MathWorks, Inc, United States)^26^.

HRV was analyzed using Kubios HRV version 2.2 software (MATLAB, Kuopio, Finland). The total period of RRi stretches were examined and the most stable segment containing 256 intervals was selected. Time domain analysis and non-linear analysis were performed in the supine position and during M-RSA only time domain analysis. The following measures were obtained for analysis in the time domain: (1) mean heart rate intervals (iRR); (2) iRR standard deviation in milliseconds (iRR standard deviation); (3) heart rate mean (HR Mean); (4) heart rate standard deviation (HR standard deviation); (5) root mean square sum of squares of the differences between the RR intervals (rMSSD); and (6) the triangular index (RRtri index).

Non-linear HRV analysis was performed from SD1 (standard deviation measuring the dispersion of points in the plot perpendicular to the line of identity), SD2 (standard deviation measuring the dispersion of points along the line of identity), alpha1 and alpha2 (respectively, short-term and long-term fluctuations of detrended fluctuation analysis), approximate entropy (ApEn) and Sample Entropy (SampEn) index^14^.

### Statistical analysis

Data were processed and analyzed using the Statistical Package for the Social Sciences—SPSS version 25.0 for Windows and were treated according to the distribution of variables by Shapiro–Wilk test. Descriptive data are reported as mean ± standard deviation, median (inter-quartile range; Q1: 25 percentile–Q3: 75 percentile) and frequency, as appropriate. The non-parametric Mann Whitney U test was used to compare the groups (Long COVID group vs Control group). For categorical variables, we applied chi-square tests (sex, smoking and caucasian ethnicity). Values of *p* < 0.05 were considered statistically significant for all tests. To analyze the power of the study sample, a post hoc calculation of the power of the sample was performed for the results of the comparison between the Long COVID groups (n = 21) and Control Group (n = 20). Two-way independent t-test was used, with α = 0.05. The effect size was determined by the mean of each group for the variable rMSSD during M-RSA (Long COVID: 14.7 ms and Control Group: 45.2 ms) and standard deviation of the groups. Thus, the value of sample power was (1 − β) = 0.999, considered a large sample power. The sample size calculation was performed using G*Power 3.1 (University of Dusseldorf, Germany).

## Data Availability

Data supporting the findings of this work are available within the paper. Data supporting the results of this study are available upon request to the corresponding author. [Andréa Lúcia Gonçalves da Silva, PT, PhD]. Data is not publicly available due they contain information that may compromise the privacy/consent of research participants”.
